# In Vitro Micropropagation of Oca (*Oxalis tuberosa* Mol.): An Important Plant Genetic Resource from the High Andean Region

**DOI:** 10.3390/plants15010062

**Published:** 2025-12-25

**Authors:** Erika Llaja-Zuta, Deyli Mailita Fernández-Poquioma, Biverly Añazco-Urbina, Angel David Hernández-Amasifuen, Jorge Alberto Condori-Apfata

**Affiliations:** Instituto de Investigación, Innovación y Desarrollo para el Sector Agrario y Agroindustrial (IIDAA), Facultad de Ingeniería y Ciencias Agrarias, Universidad Nacional Toribio Rodríguez de Mendoza de Amazonas, Calle Higos Urco 342—Ciudad Universitaria, Chachapoyas 01000, Peru; 7262322721@untrm.edu.pe (E.L.-Z.); 7223347721@untrm.edu.pe (D.M.F.-P.); biverly.anazco.epg@untrm.edu.pe (B.A.-U.); angel.hernandez@untrm.edu.pe (A.D.H.-A.)

**Keywords:** 6-benzylaminopurine, indole-3-butyric acid, ex vitro acclimatization, Andean tuber crop, germplasm conservation

## Abstract

Oca (*Oxalis tuberosa* Mol.) is an Andean crop with high nutritional and cultural value; however, its vegetative propagation makes it challenging to ensure a continuous supply of high-quality planting material. In this study, an efficient and reproducible in vitro propagation protocol was established for the oca genotype OT–001 (Amazonas, Peru), integrating shoot multiplication, rooting, and acclimatization. One-centimeter nodal segments were cultured in MS medium supplemented with 6-benzylaminopurine (BAP) or kinetin (KIN) at increasing concentrations ranging from 0.1 to 2.0 mg L^−1^. For rooting, one-centimeter shoots were transferred to MS medium supplemented with indole-3-butyric acid (IBA) or 1-naphthaleneacetic acid (NAA) at increasing concentrations ranging from 0.1 to 2.0 mg L^−1^. The variables evaluated four weeks after treatment initiation were regeneration percentage, rooting percentage, number of shoots per explant, number of roots per explant, number of nodes, and shoot length. The regeneration rate reached 100% with both BAP and KIN treatments; however, shoot proliferation was highest with 1.0 mg L^−1^ BAP, producing an average of 7.4 shoots per explant compared to 2.3 shoots in the control. Meanwhile, KIN concentrations of 0.2–0.5 mg L^−1^ promoted the development of longer shoots (up to 31.4 mm). In rooting, although the control achieved 93.3%, auxin supplementation improved root architecture. IBA at 0.1 mg L^−1^ achieved 100% rooting with the longest roots (23.9 mm), while 2.0 mg L^−1^ IBA maximized the number of roots (14.2 roots per explant). With NAA, the root systems were dense but shorter. The in vitro-regenerated plantlets exhibited 100% survival after 15 and 30 days of acclimatization in sterile agricultural soil, demonstrating the high quality of the plant material obtained. The protocol enables the production of homogeneous and vigorous plantlets throughout the year and provides a practical foundation for the ex situ conservation of oca germplasm and its commercial propagation. It also establishes the basis for advanced applications such as genetic transformation and gene editing.

## 1. Introduction

The oca (*Oxalis tuberosa* Mol.) is a traditional tuber crop from the high Andean regions that provides nutritional, cultural, and economic value to Andean communities. Its cultivation relies on a broad genetic diversity and a complex history of domestication [1,2]. In addition to its nutritional importance, oca is notable for its starch which exhibits distinctive functional properties, and for its bioactive metabolite that could enhance modern food applications. These traits, along with its adaptability across different altitudes, justify efforts to improve its propagation and ensure the year-round availability of high-quality planting material [3,4,5,6].

However, oca faces challenges that limit productivity and the safe exchange of germplasm. Vegetative propagation promotes the accumulation and transmission of viruses, which reduce vigor, yield, and quality; therefore, the use of certified, pathogen-free starting material is critical [7,8,9,10]. In addition, genetic erosion has led to a loss of diversity, and post-harvest variability in compounds such as oxalates can affect tuber quality [11,12]. These factors impact yield and quality and complicate the sanitary management of the crop, particularly under informal seed systems. Collectively, these limitations highlight the need for strategies to ensure healthy, uniform and traceable planting material, a goal for which in vitro tissue culture approaches have proven especially valuable in vegetatively propagated root and tuber crops [13,14].

In this context, in vitro plant tissue culture, particularly micropropagation, is a well-established strategy for rapid multiplication of elite material, the production of pathogen-free plants, and the year-round availability of plantlets under controlled conditions [15,16]. Likewise, the standardization of in vitro regeneration and multiplication protocols supports the ex situ conservation of native or threatened germplasm [17,18,19], and enables advanced breeding applications, from genetic transformation to gene editing [20,21,22].

Depending on the objective, apical meristems, nodal segments, leaves, roots, hypocotyls, or anthers can be used, and even protoplasts; the latter enable organogenesis or somatic embryogenesis pathways that result in clonal plants true to the original genotype [23,24,25,26]. This approach is applicable to both herbaceous and woody species. In practice, however, the morphogenic response is often highly dependent on the genotype and the composition of the medium, which requires fine-tuning of growth regulators and culture conditions for each genotype [27,28,29].

Specific evidence in oca indicates that plants can be regenerate from explants under appropriate combinations of auxins and cytokinins, and that viruses can even be eradicate through thermotherapy and in vitro chemotherapy, although the responses vary among genotypes and growing conditions [7,30,31]. These findings underscore the need for genotype-specific protocols in each oca-growing region, particularly for the poorly characterized germplasm from the Amazonas region of Peru, which currently lacks standardized in vitro micropropagation methods. Such protocols are essential for conserving regional germplasm and promoting its use in breeding and value-added initiatives.

Consequently, the objective of this study was to establish the optimal combinations of growth regulators for in vitro multiplication and rooting in an oca genotype from the Amazon region, and to evaluate its ex vitro acclimatization. The results could contribute to the sustainable use of germplasm, improve commercial production with pathogen-free material, and provide a replicable foundation for future genetic improvement programs of oca.

## 2. Materials and Methods

### 2.1. Plant Material and Aseptic Establishment

**Figure 1:**
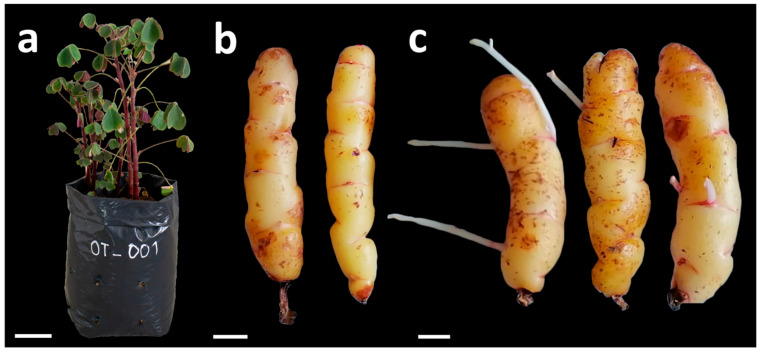
Plant material of *Oxalis tuberosa* accession OT–001 used for in vitro establishment: (**a**) cultivated donor plant (scale bar = 5 cm); (**b**) tubers harvested from cultivated plants (scale bar = 1 cm); (**c**) tubers approximately 30 days old kept at room temperature, showing sprouts used as a source of explants (scale bar = 1 cm).

The oca tubers of accession OT–001 were collected from the district of Lamud, Luya province, Amazonas region, Peru. At the Plant Molecular Biology Laboratory of the Universidad Nacional Toribio Rodríguez de Mendoza (UNTRM), the tubers were planted in polyethylene bags containing agricultural soil and established for plant growth and development (Figure 1a) and for tuber production (Figure 1b). For sprouting, tubers were kept at room temperature (20–22 °C) in the dark and at ambient relative humidity for approximately 30 days to induce bud emergence, and the emerging shoots were used as a source of explants (Figure 1c).

Initial cleaning consisted of immersion in a neutral detergent for 10 min followed by rinsing under running water for 15 min. In a laminar flow cabinet, the explants were surface disinfected with 70% (*v*/*v*) ethanol for 1 min, followed by 1% (*v*/*v*) sodium hypochlorite (NaClO) for 10 min, and three rinses with sterile distilled water. Nodal segments were cut to approximately 1 cm and cultured on half-strength Murashige & Skoog (MS) medium [32] supplemented with 1.5% (*w*/*v*) sucrose and 0.7% (*w*/*v*) agar, adjusted to pH 5.8. The cultures were incubated at 24 ± 1 °C under a light intensity of 80 μmolm^−2^ s^−1^ with a 16/8 h (light/dark) photoperiod. With this surface sterilization protocol, no microbial contamination was observed in the initial cultures of nodal explants.

### 2.2. Induction and Proliferation of Shoots

Shoots of approximately 10 cm were obtained from donor plants grown from sprouted tubers (~30 days old). Nodal segments were cut to approximately 1 cm, and explants were cultured in MS medium either without plant growth regulators (PGRs) or supplemented with independent gradients of 6-benzylaminopurine (BAP) or kinetin (KIN) at 0.1, 0.2, 0.5, 1.0, and 2.0 mg L^−1^. All media were prepared with 3% (*w*/*v*) sucrose and 0.7% (*w*/*v*) agar, adjusted to pH 5.8. Cultures were incubated at 25 ± 1 °C under a light intensity of 80 μmolm^−2^ s^−1^ with a 16/8 h (light/dark) photoperiod. After four weeks, the following parameters were evaluated: (i) regeneration percentage (explants with shoots/total explants × 100), (ii) number of shoots per explant, (iii) number of nodes per explant, and (iv) shoot length (mm). The morphogenic response was evaluated after four weeks of culture using these same variables, considering an explant as regenerated when at least one visible shoot had emerged.

### 2.3. Root Induction

Healthy explants of regenerated shoots, 1 cm in length, were transferred to rooting media: MS medium without PGRs or supplemented with independent gradients of indole-3-butyric acid (IBA) or 1-naphthaleneacetic acid (NAA) at 0.1, 0.2, 0.5, 1.0, and 2.0 mg L^−1^. All media contained 3% (*w*/*v*) sucrose and 0.7% (*w*/*v*) agar, adjusted to pH 5.8. Cultures were incubated at 25 ± 1 °C under a light intensity of 80 μmol m^−2^ s^−1^ with a 16/8 h (light/dark) photoperiod. After four weeks, the following parameters were recorded: (i) rooting percentage, (ii) number of roots per explant, and (iii) root length (mm). For acclimatization, plantlets rooted on MS medium containing 2.0 mg L^−1^ IBA were maintained for an additional four weeks on the same medium to promote root elongation, strengthening and size uniformity prior to transplanting.

### 2.4. Acclimatization of In Vitro Regenerated Plants

Rooted plantlets were removed from the MS culture vessels and gently washed with distilled water to eliminate any remaining medium. At the time of transplanting, plantlets were approximately 5 cm in height and their roots were not pruned prior to planting. The plantlets were then transplanted into pots containing sterile agricultural soil and placed directly in a growth chamber at 22 ± 2 °C under a 16/8 h (light/dark) photoperiod and 60% relative humidity, without an additional high-humidity enclosure, and were watered every two days. Survival was evaluated 15 and 30 days after transplanting.

### 2.5. Experimental Design and Statistical Analysis

The experiments were arranged in a completely randomized design (CRD). For each treatment, three explants were cultured per replicate, with 15 replicates per treatment, resulting in a total of 45 explants per experiment. The assumptions of normality and homogeneity of variances were verified using the Shapiro–Wilk test [33] and Levene test [34], respectively. Once these assumptions were met, the data were subjected to analysis of variance (ANOVA), and mean comparison were performed using Tukey’s HSD test (α = 0.05) [35]. Statistical analyses were conducted using R software (version 4.5.1 for Windows) [36], with the car [37] and agricolae [38] packages.

## 3. Results and Discussion

### 3.1. Shoot Induction and Proliferation

**Figure 2:**
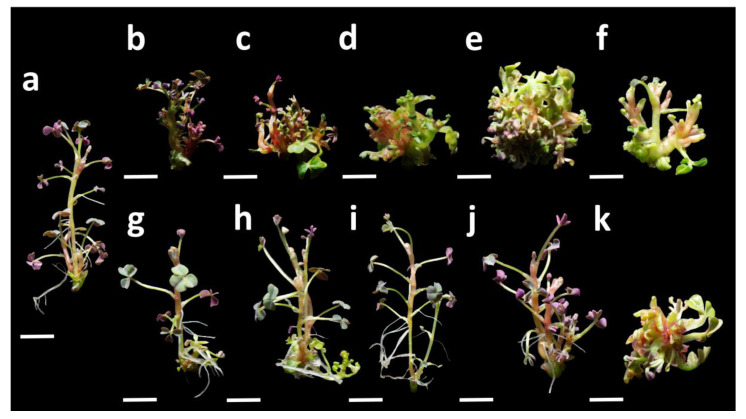
Morphogenic response of *Oxalis tuberosa* explants after 4 weeks of culture on MS medium supplemented with cytokinins. (**a**) Oca in MS medium without regulators; (**b**–**f**) Oca in MS medium + BAP at 0.1, 0.2, 0.5, 1.0, and 2.0 mg L^−1^; (**g**–**k**) Oca in MS medium + KIN at 0.1, 0.2, 0.5, 1.0, and 2.0 mg L^−1^. Compact shoot proliferation with short internodes was observed in BAP treatments, whereas KIN promoted more elongated shoots without reducing the number of nodes. Scale bar = 5 mm (all panels).

Morphogenesis occurred both in MS medium without PGRs and in media supplemented with cytokinins; however, the addition of PGRs significantly increased the regeneration rate (Table 1). While the control treatment reached 86.67%, several combinations with BAP or KIN achieved 100% regeneration, confirming the effectiveness of cytokinins in inducing sprouting in *O. tuberosa*. Regarding shoot proliferation, the best performance was observed with BAP at 1.0 mg L^−1^, which produced 7.4 ± 0.2 shoots per explant, significantly higher than the control (2.3 ± 0.1) and all other concentrations (Table 1). However, at low concentrations of BAP, the shoots exhibited shorter internodes, with lengths between 16–17 mm, and the number of nodes decreased compared to the control, indicating a compaction effect typical of BAP, which promotes shoot proliferation at expense of elongation (Figure 2a–f). With KIN, an opposite pattern was observed at moderate concentrations (0.2–0.5 mg L^−1^): explants maintained 100% regeneration with shoots reaching up to 31.4 ± 2.4 mm at 0.5 mg L^−1^, although with fewer shoots per explant than BAP at 1.0 mg L^−1^ (Figure 2g–k). At higher KIN concentrations (2.0 mg L^−1^), shoot length decreased, indicating a loss of the elongation advantage, while the number of shoots increased (5.7 ± 0.2 at 2.0 mg L^−1^).

Our results demonstrate a high basal morphogenic potential in *O. tuberosa* (86.7% in MS medium without PGRs) and confirm that the addition of cytokinins increases the regeneration rate to 100%, modifying the proliferation–elongation balance. The highest number of shoots per explant was obtained with BAP at 1.0 mg L^−1^ (7.4 ± 0.1), whereas KIN at 0.2–0.5 mg L^−1^ promoted greater shoot length. This pattern aligns with the role of cytokinins in releasing axillary buds, modulating apical dominance, and regulating the cell division-elongation ratio, processes strongly influenced by the mineral composition of the medium and the applied growth regulators [39,40].

Compared with previous studies on the in vitro multiplication of oca, our peak responses with BAP (0.2–1.0 mg L^−1^) are consistent with the pioneering work of Ochatt et al. [41], who employed a two-stage process (multiplication and rooting) and reported 8.1 shoots per explant using NAA at 0.1 mg L^−1^ + KIN at 5.0 mg L^−1^, although with compact shoots requiring an additional elongation phase. Similarly, Conner & Christey [31] demonstrated that varying sucrose concentration 30 vs. 60 g L^−1^ in MS medium did not affect the multiplication rate but influenced starch accumulation, emphasizing that shoot architecture can be decoupled from shoot number. Finally, Mejía-Muñoz et al. [42], reported high responses when combining BAP at 1.0 mg L^−1^ with NAA at 1.0 mg L^−1^, obtaining up to 9 shoots per explant from axillary buds, slightly higher than our maximum with BAP alone (7.4 shoots per explant), highlighting a cytokinin–auxin synergy modulated by the plant material and the subculture stage.

Mechanistically, the compaction phenotype observed with BAP, characterized by short internodes and fewer nodes, is consistent with enhanced activation of axillary meristems and a bias toward cell division rather than elongation. Conversely, KIN at moderate concentrations shifts the balance toward elongation and increased nodulation, traits valuable for elongation subcultures prior to rooting [39,40]. Beyond the effect of cytokinins, the in vitro response is strongly genotype-dependent from the establishment stage through multiplication, with Andean ecotypes differing in sprouting rate, elongation, and shoot architecture [43]. This variation aligns with the species domestication history and high level of polyploidy (often octoploid), which together explain the discrepancies reported among studies. Consequently, it is methodologically sound to develop genotype- or variety-specific protocols tailored to regional germplasm in Peru [43,44,45,46].

### 3.2. Root Induction

**Figure 3:**
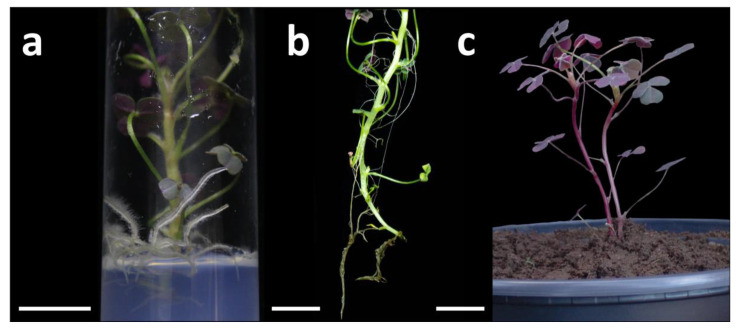
In vitro rooting and acclimatization of *Oxalis tuberosa* (accession OT–001). (**a**) In vitro-regenerated plantlet cultured on MS rooting medium supplemented with auxin, showing adventitious root formation after 4 weeks (bar = 1 cm); (**b**) plantlet removed from the flask with a well-developed root system, prior to transplanting (bar = 1 cm); (**c**) acclimatized plantlet after 30 days in a pot containing sterile agricultural soil, showing vigorous growth (bar = 3 cm).

In oca explants, in vitro rooting occurred both on MS medium without auxins and on MS medium supplemented with NAA or IBA, with percentages above 80% in all treatments and reaching 100% in MS medium containing NAA at 0.1–2.0 mg L^−1^ and IBA at 0.1–0. 2–2.0 mg L^−1^ (Table 2). Although the control exhibited high rooting (93.33%), the addition of auxins improved root system quality by increasing both the number and length of roots compared with the control (Figure 3a,b). With NAA, the rooting percentage remained stable (80–100%), with peaks at 0.1 and 2.0 mg L^−1^. The number of roots ranged from 7.4 to 9.8 per explant, with lengths between 13 and 15 mm, exceeding control but remaining below the values obtained with IBA. In practical terms, NAA promoted the formation of a dense root system of moderate length, suitable when early anchorage is prioritized. IBA produced the best root quality indicators. The highest number of roots was achieved with 2.0 mg L^−1^ (14.2 ± 0.6 roots), significantly greater than in the other treatments. For root elongation, the greatest root length was obtained at 0.1 mg L^−1^ IBA (23.9 ± 0.6 mm), followed by 0.2 and 0.5 mg L^−1^ (21.1 ± 0.6 and 19.6 ± 0.5 mm, respectively). Overall, IBA outperformed NAA in both length and the maximum number of roots per explant.

Our results show that auxin supplementation improves root architecture compared to the control, and that IBA outperforms NAA in both root elongation, at 0.1 mg L^−1^, roots reached 24 mm, and in root density, at 2.0 mg L^−1^, 14.2 roots per explant were obtained. This behavior aligns with the known properties of auxins in culture. IBA is more stable and less prone to oxidation, and it effectively promotes adventitious root initiation. At low doses, it favors the development of long, functional roots, whereas at higher doses, it increases the number of roots with slight reduction in elongation [39,40,47].

At the mechanistic level, the dose-dependent responses to IBA and NAA observed in this study are consistent with the central role of auxin signaling and distribution in controlling adventitious root formation. More stable auxins, such as IBA are known to favor sustained auxin accumulation at the base of the shoot, promoting the reprogramming of competent cells into root initials and supporting the emergence of a greater number of elongated roots [48,49]. Although molecular components were not evaluated here, the shift from longer roots at low IBA concentrations to a denser root system at higher doses aligns with general models in which auxin levels and local gradients modulate both the initiation and subsequent elongation of adventitious roots [49,50].

In oca, the sensitivity of rooting to auxins and the advisability of separating the multiplication and rooting phases have already been reported. In the protocol developed by Ochatt et al. [41], the multiplication medium (0.1 mg L^−1^ NAA + 5.0 mg L^−1^ KIN) produced approximately 50% of explants with roots after 20 days. These results are lower than our maxima of 100% with IBA 0.1–0.2 mg L^−1^ (longer roots) and IBA 2.0 mg L^−1^ (greater number of roots), reinforcing that both the dedicated rooting phase and the type of auxin define root quality. Similarly, Khan et al. [30] reported that regenerated shoots rooted in basal MS medium and could be successfully transferred to soil, which agrees with our high rooting rate in the control (93.3%) and suggests that IBA can be advantageously used to enhance root architecture (number and length) beyond that obtained with MS medium without regulators.

Likewise, when compared to multiplication protocols that combine cytokinins and auxins in oca, our results with IBA in the specific rooting phase complement previous findings. In the study by Mejía-Muñoz et al. [42], the use of BAP 1.0 mg L^−1^ + NAA 1.0 mg L^−1^ produced approximately nine shoots per explant and good root establishment. Meanwhile, Conner et al. [31] reported that increasing sucrose concentration did not affect the multiplication rate but shortened internodes, thickened tissues, and increased starch accumulation, thereby improving post-transplant performance.

Finally, it is important to consider the genotype dependence previously documented for *O. tuberosa* during establishment and multiplication. Differences among Andean ecotypes may account for part of the variability observed across studies and reinforce the need for genotype- or variety-specific protocols, particularly when defining the effective range of IBA concentrations and exposure times prior to acclimatization [43,45].

Beyond the composition of the culture medium, the physical environment during shoot multiplication and rooting (25 °C and a 16/8 h light/dark photoperiod) likely contributed to the robust morphogenic responses observed. This range of temperature and photoperiod is commonly considered optimal for in vitro morphogenesis in many species and helps to avoid both thermal and photo-induced stress while maintaining sufficient daily irradiance for growth [51,52]. In addition, several studies have shown that temperature and light regime can modulate the balance between shoot proliferation and elongation, as well as adventitious root induction, through their effects on photosynthetic activity, carbohydrate status, and endogenous hormone levels [53,54]. Although these factors were kept constant in this study to isolate the effects of growth regulators, future protocol standardization for other oca genotypes should explicitly evaluate alternative light–temperature combinations and their interactions with PGRs.

### 3.3. Acclimatization of In Vitro Regenerated Plants

The ex vitro acclimatization of *Oxalis tuberosa* achieved 100% survival at both 15 and 30 days, with vigorous plantlet growth (Figure 3c). This performance confirms that the quality of the root system obtained in vitro determines ex vitro success and that maintaining an initial controlled environment mitigates transplant shock caused by atmospheric change, facilitating the photoautotrophic transition. Similar results have been reported for other crops, highlighting that long, well-differentiated roots, together with basic control of humidity and light, significantly enhance plant establishment during acclimatization [16,17,47].

Previous studies on oca have reported that separating the multiplication and rooting phases improves plant establishment in pots or in the field. Ochatt et al. [41] achieved 100% rooting and good survival when rooting was conducted as a distinct phase prior to transplanting, while Conner et al. [31] demonstrated that increasing sucrose concentration during propagation did not affect sprouting rate but enhanced starch accumulation, resulting in improved post-transplant establishment. In our study, 100% survival was obtained in sterile agricultural soil, comparable to or exceeding these previous results, suggesting that the functional roots are developed, a simple substrate can be sufficient, provided that the initial microenvironment is properly controlled.

Although the physical, chemical, and microbiological properties of the agricultural soil used during acclimatization were not characterized in this study, the 100% survival of plantlets suggests that its structure and water-holding capacity were adequate to sustain root function and gas exchange during the transition to ex vitro conditions [55]. Substrate attributes such as texture, porosity, water and nutrient availability, and, in non-sterile systems, microbial activity or microbial metabolites are known to strongly influence the acclimation performance and subsequent growth of micropropagated plants [56]. Future work should therefore compare alternative soil or substrate types and explicitly relate their physical and biological characteristics to physiological indicators of oca plantlet establishment under ex vitro conditions.

From a physiological standpoint, micropropagated plantlets typically exhibit poorly functioning stomata and a reduced cuticle; therefore, fine-tuning photoperiod, temperature, and relative humidity are crucial during the first few weeks ex vitro. In our case, the combination of long roots obtained with low IBA concentrations (0.1–0.2 mg L^−1^) and 60% relative humidity regime favored rehydration and the restoration of stomatal control. When the goal is to maximize anchorage, a short phase with high IBA (2.0 mg L^−1^) promotes root system densification without compromising plant health, provided that evaporation and progressive ventilation are carefully managed [39,40].

In practical terms, these results support an operational flow for the oca, that includes multiplication, rooting with IBA (low concentrations for elongation or high for density), and acclimatization under a moderately controlled microenvironment. To extend this approach to the genetic diversity present in Perú, it is advisable to validate the protocol across multiple genotypes and to evaluate porous substrates (e. g. peat:perlite or coconut:perlite) or PGRs that have been shown to improve acclimatization in other crops, while considering the genotype dependence already documented for *O. tuberosa* [45,57].

Compared with previous micropropagation studies in oca, which have mostly focused on other high-Andean regions and genotypes, the present work provides a fully defined, genotype-specific protocol that integrates donor plant management, shoot induction, rooting, and acclimatization for a clone collected in the Andean highlands of the Amazonas region of northern Peru. To our knowledge, this is the first report to standardizes an in vitro propagation system for oca germplasm from this region, expanding the phenotypic and physiological information available for *Oxalis tuberosa*. By establishing effective concentration ranges of BAP, KIN, IBA, and NAA for accession OT–001, this protocol provides a practical tool for the conservation and sanitary management of regional germplasm and supports its future use in breeding and biotechnology applications.

## 4. Conclusions

This study establishes an efficient and reproducible protocol for in vitro micropropagation of oca genotype OT–001 (Amazonas, Peru), integrating multiplication, rooting, and acclimatization. In MS medium supplemented with cytokinins, BAP at 1.0 mg L^−1^ maximized proliferation, yielding 7.4 ± 0.1 shoots per explant. For rooting, a 100% response was achieved with various concentrations of IBA and NAA; however, IBA produced the best root architecture. Specifically, 0.1 mg L^−1^ generated the longest roots, 0.2–0.5 mg L^−1^ still produced markedly elongated roots, and 2.0 mg L^−1^ resulted in the highest number of roots. During the ex vitro phase, in vitro-regenerated plantlets achieved 100% survival at 15 and 30 days under controlled conditions in sterile agricultural soil, confirming the quality of the plant material produced.

Operationally, the process of propagation, rooting, and acclimatization provides a clear framework for obtaining uniform, vigorous plantlets ready for the field planting throughout the year. Given the known genotype dependence in oca, these parameters offer robust baseline ranges that can be adjusted according to variety or ecotype across different producing regions of Peru.

The results provide a practical foundation for the ex situ conservation of oca germplasm, the production of phytosanitary safe material, and the rapid multiplication of elite lines. Moreover, the establishment of consistent regeneration pathways lays the groundwork for advanced applications such as genetic transformation and editing, protoplast or somatic embryogenesis studies, as well as microtuber and synthetic seed development, thereby facilitating germplasm exchange and controlled replanting.

## Figures and Tables

**Table 1:** Effect of BAP and KIN on shoot formation from nodal explants of *Oxalis tuberosa*.

PGR	Concentration (mg L^−1^)	Percentage of Regeneration	No. of Shoots Per Explant	No. of Nodes Per Explant	Shoot Length (mm)
Control	0.0	86.7 ^c^	2.3 ± 0.1 ^g^	5.1 ± 0.3 ^a^	38.2 ± 2.0 ^a^
BAP	0.1	93.3 ^b^	3.7 ± 0.3 ^e^	4.2 ± 0.5 ^abc^	19.1 ± 0.8 ^d^
	0.2	100.0 ^a^	5.9 ± 0.2 ^c^	4.5 ± 0.3 ^ab^	17.2 ± 0.7 ^d^
	0.5	100.0 ^a^	6.9 ± 0.3 ^b^	3.1 ± 0.3 ^bc^	17.2 ± 0.5 ^d^
	1.0	100.0 ^a^	7.4 ± 0.2 ^a^	2.9 ± 0.1 ^bc^	18.3 ± 0.8 ^d^
	2.0	93.3 ^b^	4.2 ± 0.2 ^d^	2.8 ± 0.2 ^c^	18.2 ± 0.6 ^d^
KIN	0.1	100.0 ^a^	2.8 ± 0.1 ^f^	4.5 ± 0.2 ^ab^	25.5 ± 1.7 ^c^
	0.2	100.0 ^a^	3.1 ± 0.2 ^e^	5.2 ± 0.5 ^a^	26.6 ± 2.4 ^c^
	0.5	100.0 ^a^	3.5 ± 0.1 ^e^	5.7 ± 0.7 ^a^	31.4 ± 2.4 ^b^
	1.0	93.3 ^b^	4.9 ± 0.2 ^d^	5.2 ± 0.3 ^a^	26.3 ± 1.7 ^c^
	2.0	93.3 ^b^	5.7 ± 0.2 ^c^	3.1 ± 0.4 ^bc^	14.3 ± 0.7 ^e^

Means ± SE followed by different letters are significantly different according to Tukey’s test (*p* ≤ 0.05).

**Table 2:** Effect of NAA and IBA on root formation from shoot explants of *Oxalis tuberosa*.

PGR	Concentration (mg L^−1^)	Percentage of Rooting	No. of Roots Per Shoot	Root Length (mm)
Control	0.0	93.3 ^b^	4.3 ± 0.5 ^d^	8.9 ± 0.3 ^e^
NAA	0.1	100.0 ^a^	8.7 ± 0.8 ^b^	15.2 ± 0.5 ^c^
	0.2	80.0 ^d^	8.8 ± 0.5 ^b^	15.3 ± 0.3 ^c^
	0.5	86.7 ^c^	7.4 ± 0.5 ^bc^	13.3 ± 0.3 ^d^
	1.0	80.0 ^d^	6.1 ± 0.3 ^c^	13.0 ± 0.2 ^d^
	2.0	100.0 ^a^	9.8 ± 0.7 ^b^	13.3 ± 0.3 ^d^
IBA	0.1	100.0 ^a^	8.3 ± 0.4 ^bc^	23.9 ± 0.6 ^a^
	0.2	100.0 ^a^	9.9 ± 0.8 ^b^	21.1 ± 0.6 ^b^
	0.5	93.3 ^b^	8.4 ± 0.4 ^b^	19.6 ± 0.5 ^b^
	1.0	93.3 ^b^	8.9 ± 0.5 ^b^	17.0 ± 0.4 ^c^
	2.0	100.0 ^a^	14.2 ± 0.6 ^a^	16.6 ± 0.6 ^c^

Means ± SE followed by different letters are significantly different according to Tukey’s test (*p* ≤ 0.05).

## Data Availability

The datasets used and analyzed in this study are available from the corresponding author upon reasonable request.
